# Study on the Interaction Behaviors Identification of Construction Workers Based on ST-GCN and YOLO

**DOI:** 10.3390/s23146318

**Published:** 2023-07-11

**Authors:** Peilin Li, Fan Wu, Shuhua Xue, Liangjie Guo

**Affiliations:** Department of Safety Engineering, Faculty of Engineering, China University of Geosciences, Wuhan 430074, China; peilin@cug.edu.cn (P.L.); cugfun@cug.edu.cn (F.W.); 1530944@cug.edu.cn (S.X.)

**Keywords:** interaction behaviors identification, construction workers, ST-GCN, YOLO, OpenPose

## Abstract

The construction industry is accident-prone, and unsafe behaviors of construction workers have been identified as a leading cause of accidents. One important countermeasure to prevent accidents is monitoring and managing those unsafe behaviors. The most popular way of detecting and identifying workers’ unsafe behaviors is the computer vision-based intelligent monitoring system. However, most of the existing research or products focused only on the workers’ behaviors (i.e., motions) recognition, limited studies considered the interaction between man-machine, man-material or man-environments. Those interactions are very important for judging whether the workers’ behaviors are safe or not, from the standpoint of safety management. This study aims to develop a new method of identifying construction workers’ unsafe behaviors, i.e., unsafe interaction between man-machine/material, based on ST-GCN (Spatial Temporal Graph Convolutional Networks) and YOLO (You Only Look Once), which could provide more direct and valuable information for safety management. In this study, two trained YOLO-based models were, respectively, used to detect safety signs in the workplace, and objects that interacted with workers. Then, an ST-GCN model was trained to detect and identify workers’ behaviors. Lastly, a decision algorithm was developed considering interactions between man-machine/material, based on YOLO and ST-GCN results. Results show good performance of the developed method, compared to only using ST-GCN, the accuracy was significantly improved from 51.79% to 85.71%, 61.61% to 99.11%, and 58.04% to 100.00%, respectively, in the identification of the following three kinds of behaviors, throwing (throwing hammer, throwing bottle), operating (turning on switch, putting bottle), and crossing (crossing railing and crossing obstacle). The findings of the study have some practical implications for safety management, especially workers’ behavior monitoring and management.

## 1. Introduction

The construction industry has been identified as one of the most hazardous industries. And, the nature of construction projects lead to a high incidence of accidents. The interaction between man-machine, man-material, man-environments makes it complex for safety management on construction sites [1]. Managers have found that construction workers’ unsafe behaviors were an important cause of a series of accidents on construction sites [2]. According to statistics, nearly 80% of construction accidents are caused by unsafe behaviors of workers [3], and 20.6% of fatal industrial workplace accidents in the European Union occurred on the construction site [4]. One important way to prevent accidents is real-time monitoring and managing of those unsafe behaviors. Thurs, behavior-based safety (BBS) is considered as a promising approach to managing unsafe behaviors on construction sites. BBS requires observing and identifying unsafe behaviors on sites and then directly providing feedback to the workers [5,6]. The traditional way to realize it is manual inspection, which requires a lot of manpower and material resources but has non-significant effects [7].

In recent years, with the rapid development of artificial intelligence technology, construction industry practitioners have begun to realize its potential in improving construction safety management, especially in monitoring and managing construction workers’ unsafe behaviors. Many automated technologies have been proposed to monitor the behaviors of construction workers on construction sites to improve the efficiency and accuracy of unsafe behavior management [8,9,10,11,12]. The most popular way of detecting and identifying workers’ unsafe behaviors is the computer vision-based intelligent monitoring system, which could detect and identify humans or objects in two-dimensional images.

However, most existing research or products focused only on the workers’ behaviors (i.e., motions) recognition in construction sites and very limited studies considered the interaction between man-machine, man-material, or man-environments. For application, those interactions are very important for judging whether the workers’ behaviors are safe or not, from the standpoint of safety management. For example, suppose throwing a hammer is an unsafe behavior on the construction site, if a worker throws rubbish (e.g., a beverage bottle) using very similar motions, it is very difficult to judge whether the worker’s behavior is safe only based on the motion recognition result. Therefore, identifying unsafe interactions between man-machine/material is necessary and more meaningful, which could provide more direct and valuable information for safety management. To achieve the above goal, it not only needs to recognize the motion and objects, but also needs to detect the interaction. In other words, it needs to make decision rules, which is used to automatically judge whether unsafe interactions between man-machine/material occur.

Considering the importance of identifying construction workers’ unsafe interaction between man-machine/material and the limitations of existing research, this study aims to develop a method of identifying construction workers’ unsafe behaviors, i.e., unsafe interaction between man-machine/material, based on ST-GCN (for motion recognition) and YOLO (for objects, including safety signs, and detection). In this study, two trained YOLO-based models were, respectively, used to detect safety signs in the workplace, and objects that interacted with construction workers. Then, an ST-GCN model was trained to detect and identify construction workers’ behaviors. Lastly, decision rules were made, and the algorithm was developed to detect whether unsafe interactions between man–machine/material exist.

## 2. Related Works

### 2.1. Motions Recognition

For motion recognition, motion capture is the foundation and the popular computer vision-based motion capture technologies are human posture estimation algorithms such as OpenPose [13] and RGB-D sensors based technology such as Azure Kinect DK (Microsoft, Redmond, VA, USA) [14]. Despite the RGB images could be affected by light, background, imaging conditions [15], the skeletal data still can be estimated and extracted. In addition, the skeletal sequence provides only a small number of joint positions for human motion trajectories, so it has the advantage of low computational and storage requirements [16]. For motion recognition based on motion capture data, deep learning is the mostly used method, in which three different directions are derived through different joint node data processing methods, namely convolutional neural networks (CNN), long short-term memory networks (LSTM), and graph convolutional networks (GCN). The above have been widely used in detecting and identifying worker’s behaviors. Fang et al. [17] integrated Mask R CNN to identify individuals crossing structural supports. Guo et al. [18] established a 3D skeleton-based action identification method using LSTM to help automatically monitor whether safety belts are properly secured on site. Tian et al. [19] used GCN to propose a graph structure-based hybrid deep learning method to achieve the automatic classification of large-scale project safety hazard texts. Yan et al. [20] proposed a new deep learning method, spatial–temporal graph convolutional network (ST-GCN), which has the advantage of simultaneously capturing spatial and temporal information. It takes advantage of the fact that skeletons are represented by graphs rather than 2D or 3D grids, and it has achieved great success in the field of action identification. Cao et al. [21] proposed an improved ST-GCN method for recognizing unsafe mining behaviors, and achieved good performances on both public datasets and their own constructed datasets. In addition, some researchers have also made improvements based on the ST-GCN model [22,23]. Many studies have shown that ST-GCN has great potential in motion recognition.

### 2.2. Object Recognition

As mentioned above, it is more meaningful to detect and identify unsafe interactions between man–machine/material, in which the object (i.e., machine/material) recognition is also necessary. In the aspect of object recognition, a number of methods have been proposed, and detection accuracy has soared since deep learning became popular. There are mainly two types of object detection methods, one is Region Proposal based methods, and the other is the end-to-end method. The most representative of Region Proposal-based methods is the R-CNN series, including R-CNN [24], Fast R-CNN [25] and Faster R-CNN [26]. R-CNN series use region proposal methods to first generate potential bounding boxes in the image, and then run classifiers on these proposed boxes. These methods have obvious disadvantages, slow processing speed and complex pipelines that are difficult to optimize. YOLO (You Only Look Once) [27] and SSD (Single Shot MultiBox Detector) [28] are end to end methods. Compared with the R-CNN series, the YOLO method has obvious advantages, faster, more accurate and simpler, a single convolutional network could simultaneously predict multiple bounding boxes and class probabilities for these boxes. Therefore, YOLO has been widely used in the application. Sun et al. [29] improved the YOLO v5 to detect tailings ponds from high-resolution remote sensing images. Gallo et al. [30] applied YOLO v7 in weeds and crop detection and achieved better performance than the other YOLO versions. Kolpe et al. [31] used YOLO algorithm to identify masks and social distancing, eliminating the need for manual monitoring systems. Zhao et al. [32] used the advanced YOLO v4 algorithm to identify unsafe shipborne mooring and unmooring operation behaviors. Xiao et al. [33] used the YOLO v5 to monitor abnormal behaviors in substations. For the application in construction site, Hayat et al. [34] used YOLO v5 to detect safety helmets on construction sites and showed excellent detection performance even in low light conditions. Ferdous et al. [35] detected personal protective equipment on construction sites based on YOLO family’s anchor-free architecture, YOLOX, and found. YOLOX yields the highest mAP of 89.84% among the other three versions of the YOLOX. Wang et al. [36] used YOLO v5 to detect personal protective equipment on construction sites and found that YOLO v5x has the best mAP (86.55%), and YOLO v5s has the fastest speed (52 FPS) on GPU in a dedicated high-quality dataset. He et al. [37] used YOLOv5-based automatic identification to identify reflective clothing, and results showed the average accuracy reaches more than 80%, which is capable of meeting the actual needs.

### 2.3. Summary

The above indicates that technologies in motion recognition or object recognition are quite mature and have been widely used in construction workers’ unsafe behaviors management. However, the methods based on motion recognition or object recognition cannot provide enough valuable information for the identification of interaction behaviors. At present, the ways to identify the interaction between man–machine/material in construction sites are mainly integrating computer vision with natural language processing [38,39]. For example, Zhang et al. [40] proposed an identification method that inferred construction workers’ hazards through text classification of the detected construction scene graphs with specifications. Their method achieved a good performance at identifying unsafe behaviors with simple physical contact objects, but less consideration was given to complex motions. Furthermore, their method needs to extract regulatory documents and encode them in a computer-processable format, which requires a manual operation, which may be time-consuming, expensive, and error-prone.

This study elaborated the current research on the identification of unsafe behaviors at construction sites from three directions: motion recognition, object recognition, interaction recognition. And, it provided an overview of related research, as shown in Table 1.

Based on the above, most of existing research or products focused only on the workers’ behaviors (i.e., motions) recognition or object recognition, very limited research considered the interaction between man–machine/material. Considering the importance of identifying construction workers’ unsafe interaction between man–machine/material and the limitations of existing research, this study contributes a method that combines object recognition with motion recognition, which is very important for interaction identification. Furthermore, decision rules were made, and the algorithm was developed to judge whether the workers’ interaction behaviors are safe or not. The findings of the study could have some practical implications for safety management, especially workers’ behavior monitoring and management.

## 3. Methods

### 3.1. Unsafe Behaviors Selection

**Figure 1:**
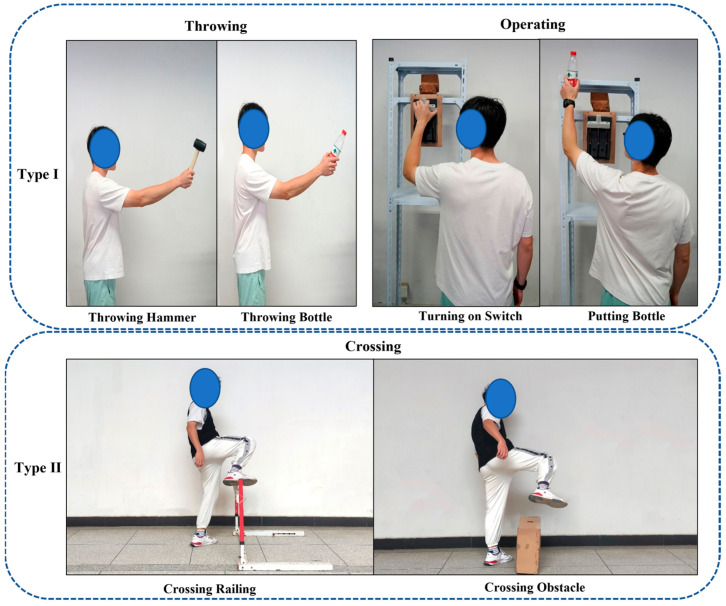
Representations of the selected behaviors.

Based on our on-site investigation, the construction workers’ unsafe interaction between man–machine/material falls into two groups: the unsafe physical contact with machine/material (Type I) and no physical contact but unsafe distance to machine/material (Type II). This paper selected six behaviors (see Table 2 and Figure 1), throwing (throwing hammer (TH), throwing bottle (TB)), operating (turning on switch (TS), putting bottle (PB)) and crossing (crossing railing (CR), and crossing obstacle (CO)), which covers above two types and are used as the experimental tasks to collect training and testing data. This study assumes that the selected the following behaviors, Throwing Hammer, Turning on Switch, and Crossing Railing are unsafe behaviors, which are prohibited. The other three behaviors, Throwing Bottles, Putting Bottles, and Crossing Obstacles are safe behaviors, but have similar features in interacted with objects or motion characteristics with the above unsafe behaviors, which are used to test the performance of the identification methods.

### 3.2. Unsafe Behaviors Identification Based on YOLO and ST-GCN

#### 3.2.1. Motion Capture

**Figure 2:**
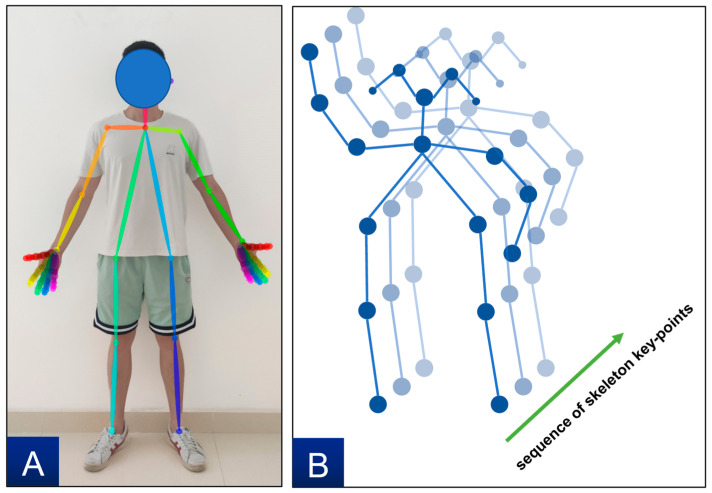
OpenPose COCO model (**A**) and sequence of skeleton keypoints (**B**).

As mentioned above, motion capture is the foundation of recognition, one of the popular computer vision-based human posture estimation algorithms is OpenPose. We utilized OpenPose for real-time 2D pose estimation from images or videos [46]. This method effectively provides position coordinates of 2D human skeletal keypoints for multiple individuals from images. OpenPose offers three pose models: MPI (15 keypoints), COCO (18 keypoints), and BODY_25 (25 keypoints), and these models differ in the number of keypoints [47]. This study used the COCO model, as shown in Figure 2A. The collected video was processed using the OpenPose algorithm to obtain human body keypoints for each frame, with keypoints connected in a fixed order. Then the human skeleton diagrams chronologically for all frames were arranged to obtain human skeleton sequence diagram, as shown in Figure 2B. In addition, OpenPose was also adopted to capture the motion of certain body parts (e.g., hands), to get more detailed motion information (e.g., the coordinates of 21 keypoints of each hand).

#### 3.2.2. ST-GCN Algorithm

Spatial Temporal Graph Convolutional Networks (ST-GCN) is the first to apply graph convolution network (GCN) to skeleton-based motion recognition tasks. ST-GCN constructs a skeleton spatial temporal graph of the skeleton keypoints sequence obtained by OpenPose, and a skeleton spatiotemporal graph G=(V,E) is obtained, as shown in Figure 2B. Where $V=\{v_{ti}|t=1,2\cdots T,i=1,2\cdots N\}$ where t represents the total number of frames of the video and i represents the number of keypoints of bones in the human body. E is composed of skeleton edges in skeleton space-time diagram, which includes two parts. The first part is the skeleton edges formed by two adjacent skeleton points in space, which is $E_{S}=\{v_{ti}v_{tj}|(i,j)\in H\}$, where H is a group of naturally connected human joints. The second part is that the skeleton edge formed by two identical skeleton points in time is composed of two subsets, which is $E_{F}=\{v_{ti}v_{(t+1)i}\}$.

**Figure 3:**
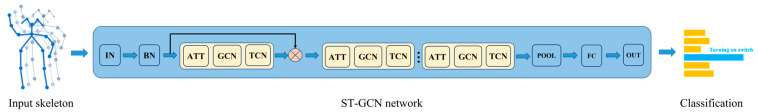
ST-GCN network structure.

As shown in Figure 3, ST-GCN processes spatial temporal skeleton graph data through multiple spatial temporal convolution modules. The basic module of spatial temporal convolution mainly consists of a temporal convolution layer and a spatial convolution layer. The network structure is composed of nine layers of basic modules with a spatial temporal convolution kernel size of 3 × 9. Each ST-GCN unit uses feature residual fusion mode to achieve cross-region feature fusion to increase the learning ability of the model. And, each ST-GCN unit adopts a dropout probability of 0.5 to reduce the risk of model overfitting. Finally, the generated feature vector is fed to SoftMax classifier to output motion classification.

#### 3.2.3. Objects Detection Technology

In this study, YOLO v5 was adopted for objects detection, which is an advanced object detection algorithm with important improvement in accuracy and speed compared to the previous YOLO versions (YOLO v1 [27], YOLO v2 [48], YOLO v3 [49], and YOLO v4 [50]). The YOLO model was trained to perform object detection from the captured videos and output the class, coordinates, and confidence.

**Figure 4:**
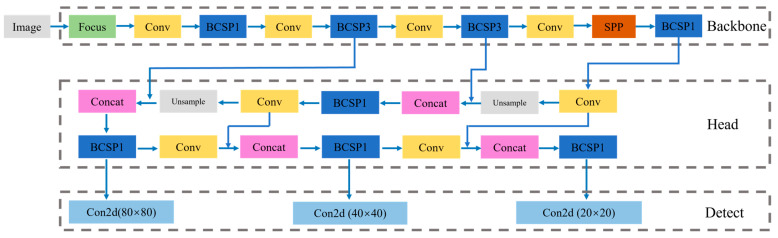
YOLO network structure.

YOLO is mainly composed of four modules: input module, backbone module, head module, and detection module, as shown in Figure 4.

(1) Input module includes Mosaic data enhancement, image size processing, and adaptive anchor frame calculation. All YOLO algorithms need to transform the size of the input image into a fixed size, and then send it into the detection model for training. The standard size of the designed image in this paper is 640 × 360 × 3.
(2) Backbone module is a kind of convolutional neural network, including Focus structure and CSP structure, which aggregates and forms image features with different image granularity. After the input image, the focus slice operation is used to extract the features more fully. At the same time, the CSPNet structure, which can extract rich features.
(3) Head module adopts the structure of FPN+PAN. FPN is top-down, and the information is transferred and fused by means of up-sampling to obtain the predicted feature map. PAN uses a bottom-up feature pyramid.

#### 3.2.4. Identification of Interaction Behaviors

Type I behaviors were identified as flows:

Step One: objects detection. YOLO v5 model was trained, and then was used to detect all the objects in each frame of the video, the object’s information, including classes of the objects, coordinates (coordinates of the upper left and lower right corners of the bounding box), and confidence level can be obtained. The detected objects include all the machines, tools, materials, safety signs, etc. contained in the image.

Step Two: motion capture and recognition. OpenPose was adopted to capture the worker’s motions, and the skeleton time sequence data, including the coordinates of 18 keypoints of the body can be obtained. In addition, when the workers perform Type I behaviors selected in this study, the body part that interacts with objects is the left or right hand. So, the skeleton time sequence data, including the coordinates of four keypoints of each hand will be specially collected. ST-GCN was trained and then used to recognize the workers’ motions, which provides the predicted probability of each motion.

Step Three: interaction behaviors identification

For Type I behaviors, whether the interaction between man-objects occurs can be judged by whether the hand keypoints are within the range formed by the bounding box. If the hand keypoints are within the range, class of objects and confidence level of objects and four hand keypoints will be recorded.

This study introduced the consideration of the number of interactions, i.e., how many times the interaction occurs. Because of the complexity of construction workers’ motions and to prevent misidentification caused by miscontact between human and machine/material, we also introduced the consideration of continuity of man-machine/material contact, i.e., the last time (number of frames) of continuous contact. The number of the frame will be recorded, which man–machine/material contact occurs.

For Type I motion identification, the discriminant parameter of each video is calculated as follows:

(1) $$P=\left\{{P_{i}|P}_{1},P_{2},P_{3}\cdots P_{n}\right\},$$

where $P_{i}$ represents the predicted probability of motion obtained by ST-GCN, and n represents the number of motions.

(2) $$C_{i}=\frac{\sum_{j=1}^{j=t_{i}}{C_{O_{i}}}_{j}\cdot C_{B_{j}}}{t_{i}}\cdot \frac{\frac{t_{i}}{TVF}\cdot w_{1}+\sum_{j=2}^{j=t_{i}}\frac{t_{i}-1}{{S_{i}}_{j}-{S_{i}}_{j-1}}\cdot w_{2}}{2},$$

(3) $$C=\left\{C_{i}|C_{1},C_{2}\cdots C_{m}\right\},$$

where $C_{i}$ represents confidence level of each object that interacted with the person, and m represents the number of objects. $t_{i}$ represents the number of interactions with $i_{th}$ object. ${C_{O_{i}}}_{j}$ and $C_{B_{j}}$ represent the confidence of the interaction object and the confidence of the left of right ankle keypoints of each interaction. TVF represents the total video frames. ${S_{i}}_{j}$ represents the frame number of the $j_{th}$ interaction. $w_{1}$ and $w_{2}$ represent the weights of the times of interactions and continuity of interactions, respectively.

(4) $$M_{i}=P_{i}\cdot w_{3}+C_{j}\cdot w_{4},$$

where $w_{3}$ and $w_{4}$ are weights of the motion and object, respectively.

(5) $$M=\max\left\{M_{i}|M_{1},M_{2},M_{3}\cdots M_{n}\right\},$$

where M represents the motion corresponding to $\max\;\left(M_{i}\right)$, (e.g., if $M=M_{2}$, $M_{2}$ represents throwing hammer, the result of behaviors identification is throwing hammer).

For each motion, the motion prediction probability is only multiplied by the corresponding object, e.g., the prediction probability of throwing hammer $P_{i}$ is only multiplied by the object confidence level of hammer $C_{i}$.

For Type II behaviors, whether the interaction between man–objects occurs can be judged by relative space position relations between body part and objects. Taking Crossing Railing (CR) and Crossing Obstacle (CO) as examples, this study firstly calculates the line function of the railing/obstacle based on the detection results of YOLO.

(6) $$f\left(x,y\right)=\frac{\frac{\sum_{i}^{n}{conv}_{i}\times {y_{1}}_{i}}{\sum_{1}^{n}{conv}_{i}}-\frac{\sum_{i}^{n}{conv}_{i}\times {y_{2}}_{i}}{\sum_{1}^{n}{conv}_{i}}}{\frac{\sum_{i}^{n}{conv}_{i}\times {x_{2}}_{i}}{\sum_{1}^{n}{conv}_{i}}-\frac{\sum_{i}^{n}{conv}_{i}\times {x_{1}}_{i}}{\sum_{1}^{n}{conv}_{i}}}\left(x-\frac{\sum_{i}^{n}{conv}_{i}\times {x_{2}}_{i}}{\sum_{1}^{n}{conv}_{i}}\right)+\frac{\sum_{i}^{n}{conv}_{i}\times {y_{1}}_{i}}{\sum_{1}^{n}{conv}_{i}}-y,$$

where n represents the total video frames of each video, (${x_{1}}_{i}$ ${y_{1}}_{i}$) and (${x_{2}}_{i}$ ${y_{2}}_{i}$) represents upper-left and lower-right coordinates of the object (i.e., bounding box detected by YOLO) for each frame of the video.

Secondly, whether the interaction between man-objects occurs can be judged by the change of left/right ankle’s coordinates.

(7) $$Q_{j_{i}}=f\left(x_{{{ankle}_{j}}_{i-1}},{y_{{ankle}_{j}}}_{i-1}\right)\cdot f\left(x_{{{ankle}_{j}}_{i}},{y_{{ankle}_{j}}}_{i}\right),i=2,3\cdots n,j=1,2,$$

(8) $$Q_{j}=\left\{Q_{j_{1}},Q_{j_{2}}\cdots Q_{j_{n}}\right\},$$

where j represents the left/right ankle, $\left(x_{{{ankle}_{j}}_{i}},{y_{{ankle}_{j}}}_{i}\right)$ represents the coordinates of left/right ankle. If ${\exists Q_{j}}_{i}\in Q_{j},{Q_{j}}_{i}<0$, the interaction between man–objects occurs.

The discriminant parameter of each video is calculated as follows:

(9) $$P=\left\{{P_{i}|P}_{1},P_{2},P_{3}\cdots P_{n}\right\},$$

where $P_{i}$ represents the predicted probability of motion obtained by ST-GCN, and n represents the number of motions.

(10) $$C_{i}=\frac{\sum_{j=1}^{j=t_{i}}{C_{O_{i}}}_{j}\cdot C_{B_{j}}}{t_{i}},$$

(11) $$C=\left\{C_{i}|C_{1},C_{2}\cdots C_{m}\right\},$$

where $C_{i}$ represents confidence level of each object that interacted with the person, and m represents the number of objects. ${C_{O_{i}}}_{j}$ and $C_{B_{j}}$ represent the confidence of the interaction object and the confidence of the left of right ankle keypoints of each interaction.

(12) $$M_{i}=P_{i}\cdot w_{3}+C_{j}\cdot w_{4},$$

where $w_{3}$ and $w_{4}$ are weights of the motion and object, respectively.

(13) $$M=\max\left\{M_{i}|M_{1},M_{2},M_{3}\cdots M_{n}\right\},$$

where M represents the motion corresponding to $\max\;\left(M_{i}\right)$ (e.g., if $M=M_{2}$, $M_{2}$ represents crossing railing, the result of behaviors identification is crossing railing).

For each motion, the motion prediction probability is only multiplied by the corresponding object, e.g., the prediction probability of crossing rail $P_{i}$ is only multiplied by the object confidence level of rail $C_{i}$.

#### 3.2.5. Risk of Behaviors Evaluation Based on Safety Sign Recognition

**Figure 5:**
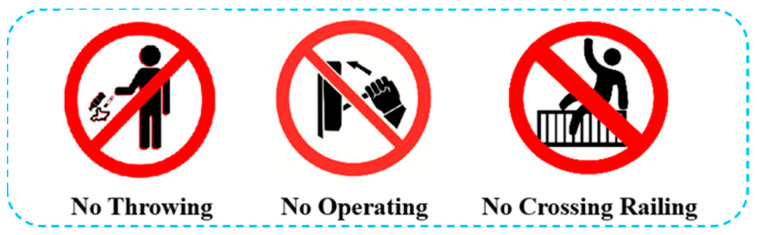
Safety signs recognized in this study.

After behavior identification, its risk should be evaluated according to the safety management and relevant regulations. This study tried to detect and recognize the safety signs in the workplace (see Figure 5), and then extract its meaning for risk evaluation. If the behavior is prohibited according to the safety signs, and corresponding safety signs were detected in the same workplace, then that behavior will be automatically judged as unsafe behavior.

### 3.3. Experiment Design

An experiment was designed and conducted to collect a large amount of motion data of simulated construction workers’ behaviors, which was used for training and testing models.

#### 3.3.1. Participants

Fourteen healthy young males (age 21.36 ± 4.64 years; height 179.62 ± 4.86 cm; weight 75.79 ± 4.69 kg) volunteered to participate in this study. Each participant signed an informed consent form on the experimental protocol.

#### 3.3.2. Experimental Equipment and Task

**Figure 6:**
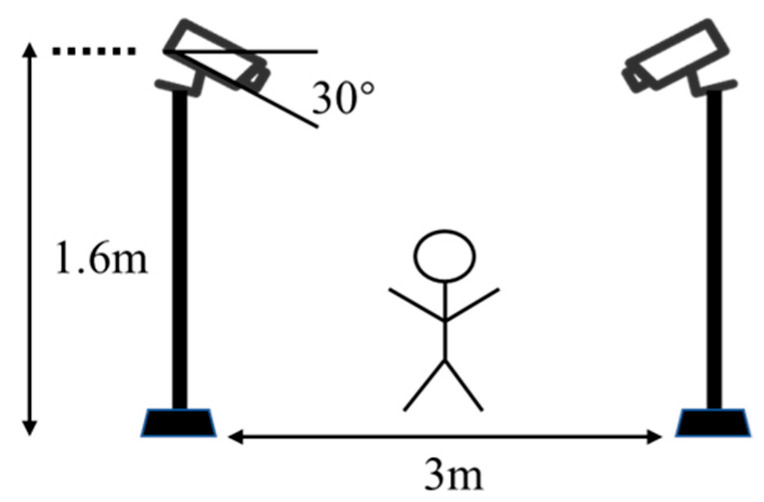
Experimental apparatus and settings.

In this study, two cameras were used to collect video data, with a recording resolution of 1920 × 1080 at a frequency of 24 FPS. The two cameras, with 30 degrees downward, were placed on the left and right of the participant (see Figure 6). Moreover, one hammer (240 mm long), two beverage bottles (550 mL capacity, 220 mm high), one electric switch (253 mm × 153 mm × 90 mm), one railing (1050 mm wide, 600 mm high), and a cardboard rectangle box (600 mm × 200 mm × 400 mm, used as obstacle) were used as the objects that interacted with participants. Each participant was asked to perform six simulated construction worker’s behaviors (see Table 2 and Figure 1) in sequence, each task was repeated five times with both hands. Video data was collected in the process.

### 3.4. Training of the Model

After collecting the experimental data, the training and testing of the YOLO and ST-GCN network models were carried out on a laptop computer. The configuration parameters of the software and hardware platform in this study are shown in Table 3.

For YOLO network model training, the dataset was divided in the randomly partitioned dataset into a training set and a validation set in a ratio of 8:2. The batch__size, was set to 32, epoch was set to 50, weight_decay was set to 0.0005, and the initial weight model file was YOLOv5s.pt. For ST-GCN network training, the dataset was divided into a training set, a validation set and a testing set in a ratio of 6:2:2. The batch__size was set to 32, the epoch was set to 100, the weight_decay was set to 0.0005, the base_lr was set to 0.001, and the learning rate was adjusted to decay every 20 rounds, where the decay rate was 0.1.

The performance of the models was tested using the following methods. For binary classification, Precision, Recall, and $F_{1}-Score$ were taken as metrics. The equations for these metrics are shown as follows.

(14) $$Precision=\frac{TP}{TP+FP},$$

(15) $$Recall=\frac{TP}{TP+FN},$$

(16) $$F_{1}=2\times \frac{Precision\times Recall}{Precision+Recall},$$

where TP, FP, and FN are abbreviations for True Positive, False Positive, and False Negative.

For multi-class classification, macro-average was used to evaluate the model. The formulas are shown as follows.

(17) $$\mathrm{Precision}=\frac{1}{n}\sum_{i=1}^{n}Precision_{i},$$

(18) $$\mathrm{Recall}=\frac{1}{n}\sum_{i=1}^{n}Recall_{i},$$

(19) $$F_{1}=\frac{1}{n}\sum_{i=1}^{n}F_{1_{i}}.$$

## 4. Results

### 4.1. Data Collection

For the video shooting, we shot 5040 videos in total, as shown in Table 4 in detail.

### 4.2. YOLO Training Results

Input the training set photos into the YOLO neural network for training, and the results are shown in Table 5. The results show Precision and mAP@0.5 of all objects and safety signs were close to 1.00, and Recall was 1, indicating the trained YOLO model meets the requirements of recognition of objects and safety signs in the experimental videos.

### 4.3. Results of Behaviors Identification Only Based on ST-GCN

In order to compare the differences in performance between the ST-GCN method alone and the proposed YOLO-ST-GCN method, this paper first used only the ST-GCN method to recognize the above two types of behaviors, and the results were as follows.

#### 4.3.1. Results of Type I Behaviors Identification Only Based on ST-GCN

**Figure 7:**
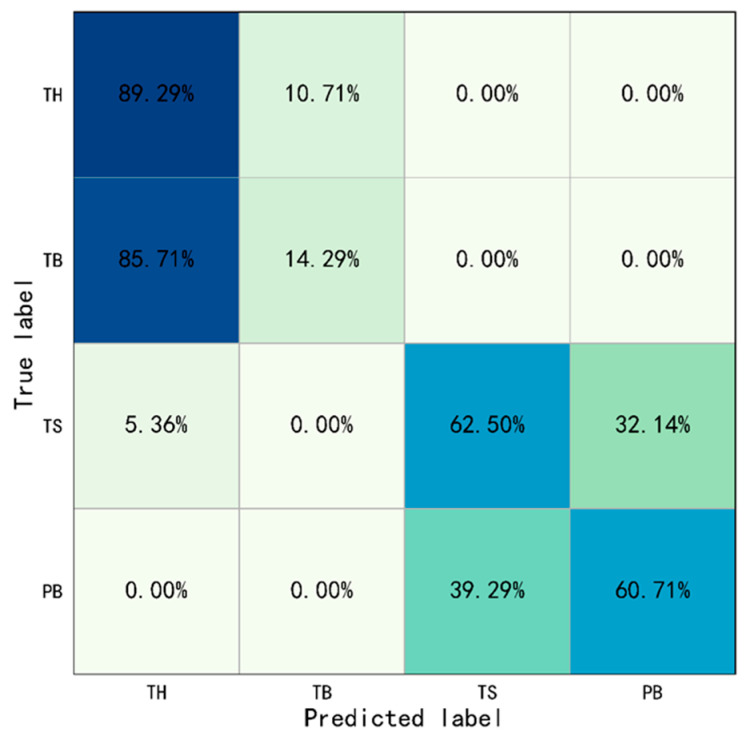
Confusion matrix of Type I behaviors identification only based on ST-GCN (TH: Throwing Hammer, TB: Throwing Bottle, TS: Turing on Switch, PB: Putting Bottle).

This study selected the weight model with the best performance on the validation set for Type I behaviors and tested it on the test set. The prediction results were then drawn into a confusion matrix, as shown in Figure 7. The accuracy of Type I behavior identification based only on ST-GCN was shown in Table 6.

The results show the overall identification accuracy of Type I behaviors were 56.70%, and the overall accuracy of Throwing and Operating were 51.79% and 61.61%, respectively. The accuracy of throwing hammer, throwing bottle, turning on switch, and putting bottle were 89.29%, 14.29%, 62.50% and 60.71%, respectively. Especially since, the rate of which the throwing bottle was misidentified as throwing hammer and was 85.71%. The evaluation indicators were also calculated: $Precision=0.58,\;Recall=0.57,\;and\;F_{1}-score=0.53$. The above results indicated the performance of only based on ST-GCN was very poor, which means that it is difficult to recognize the Type I behaviors only based on ST-GCN.

#### 4.3.2. Results of Type II Behaviors Identification Only Based on ST-GCN

**Figure 8:**
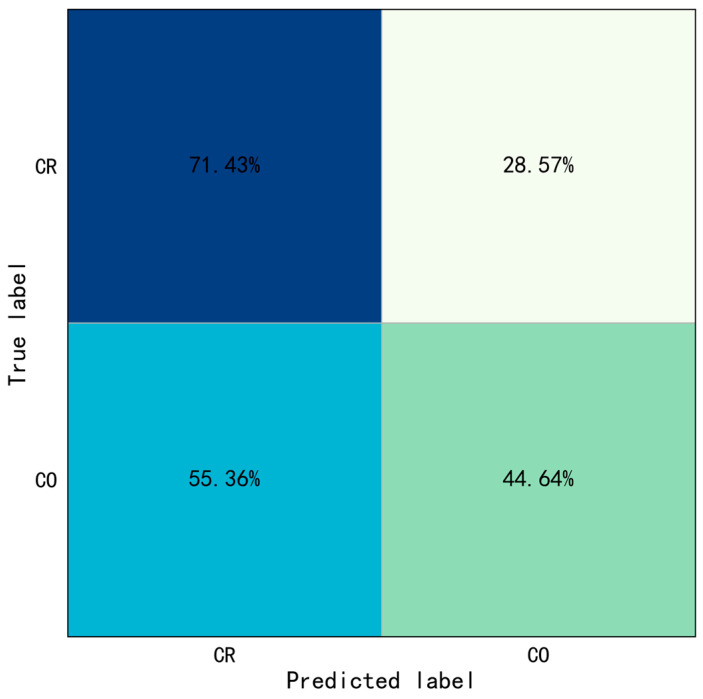
Confusion matrix of Type II behaviors identification only based on ST-GCN (CR: Crossing Railing, CO: Crossing Obstacle).

Similarly, this study selected the weight model with the best performance on the validation set to test the test set, and then draw the prediction results into a confusion matrix, as shown in Figure 8. The accuracy of Type II behaviors identification only based on ST-GCN was shown in Table 7.

The results show the overall identification accuracy of Type II behaviors was 58.04%, the accuracy of crossing railing and crossing obstacle was 71.43% and 44.64%, respectively. Especially since, the rate of which for the crossing obstacle was misidentified as crossing railing was 55.36%. The crossing railing was set as positive samples, crossing obstacle was set as negative samples. The evaluation indicators were also calculated: $Precision=0.56,\;Recall=0.71,\;and\;F_{1}-score=0.63$. The above results indicated the performance of only based on ST-GCN was poor, which means that it is difficult to recognize the Type II behaviors only based on ST-GCN.

### 4.4. Results of Behaviors Identification Based on YOLO-ST-GCN

#### 4.4.1. Results of Type I Behaviors Identification Based on YOLO-ST-GCN

**Figure 9:**
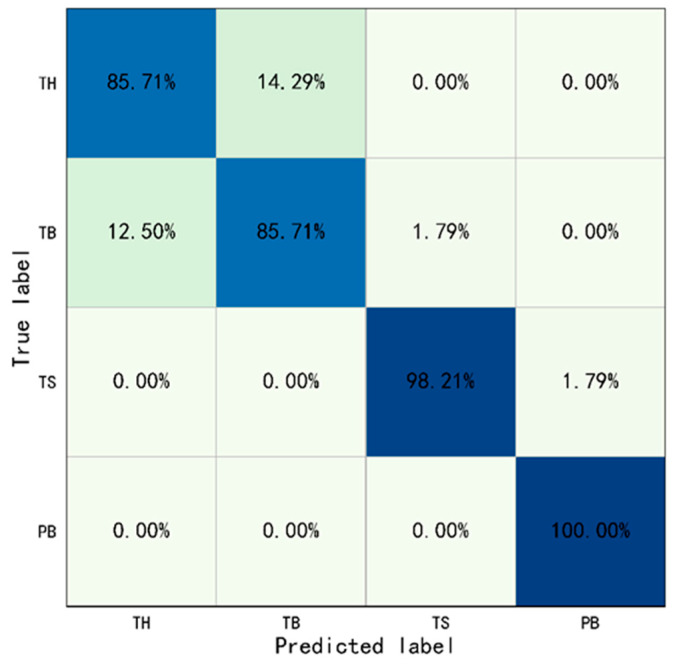
Confusion matrix of Type I behaviors identification based on YOLO-ST-GCN (TH: Throwing Hammer, TB: Throwing Bottle, TS: Turing on Switch, and PB: Putting Bottle).

For the Type I behavior, this study set $w_{1}$ = 0.4, $w_{2}$ = 0.6, $w_{3}$ = 0.6, and $w_{4}$ = 0.4. The identification results were drawn into a confusion matrix, as shown in Figure 9. The accuracy of Type I behaviors identification based on YOLO-ST-GCN was shown in Table 8.

The results show, the overall identification accuracy of Type I behaviors was 92.41%, and the overall accuracy of Throwing and Operating was 85.71% and 99.11%. The accuracy of throwing hammer, throwing bottle, turning on switch, and putting bottle were 85.71%, 85.71%, 98.21%, and 100.00%, respectively. Especially since, the rate of which for the throwing hammer was misidentified as throwing bottle and was 14.29%, and throwing bottle was wrongly identified as throwing hammer and was 12.50%. And, almost all Operating behaviors were identified correctly, with only 1.79% of the turning on switch was misidentified as putting bottle. The crossing railing was set as positive samples, crossing obstacle was set as negative samples. The evaluation indicators were also calculated: $Precision=0.92,\;Recall=0.92,\;and\;F_{1}-score=0.92$. The above results indicated that most of the Type I behaviors can be identified correctly based on YOLO-ST-GCN and the accuracy was improved greatly compared with only based on ST-GCN.

#### 4.4.2. Results of Type II Behaviors Identification Based on YOLO-ST-GCN

**Figure 10:**
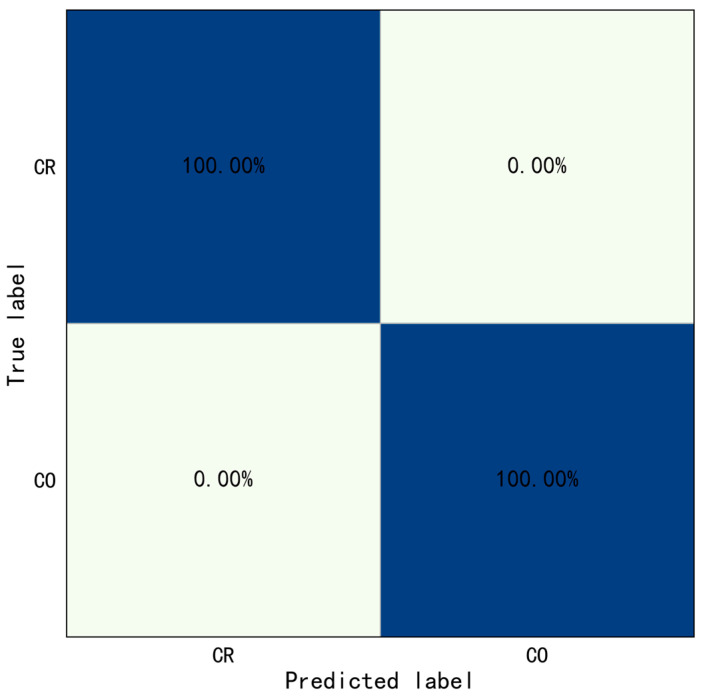
Confusion matrix of Type II behaviors identification based on YOLO-ST-GCN (CR: Crossing Railing, CO: Crossing Obstacle).

For Type II behaviors, this study set $w_{3}$ = 0.4 and $w_{4}$ = 0.6. The identification results were drawn into a confusion matrix, as shown in Figure 10. The accuracy of Type II behaviors identification based on YOLO-ST-GCN was shown in Table 9.

The results show the overall identification accuracy of Type II behaviors was 100.00%, and the accuracy of crossing railing and crossing obstacles were both 100.00%. The crossing railing was set as positive sample, crossing obstacle was set as negative sample. The evaluation indicators were also calculated: $Precision=1.00,\;Recall=1.00,\;and\;F_{1}-score=1.00$. The above results indicated that all the Type II behaviors can be identified correctly based on YOLO-ST-GCN, and the accuracy was considerably improved compared with only those based on ST-GCN.

### 4.5. Results of Behaviors Risk Evaluation Considering Safety Signs Identification

**Figure 11:**
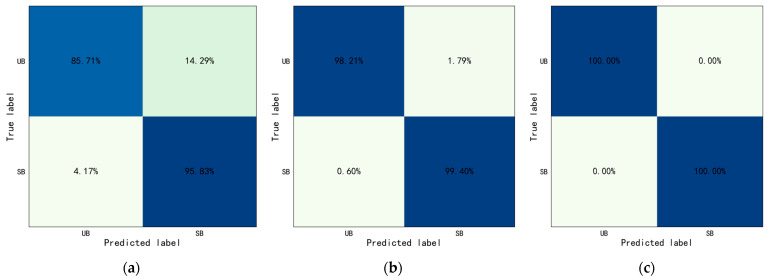
Confusion matrix of behaviors risk evaluation considering safety signs identification (UB: unsafe behavior and SB: safe behavior). (**a**) No Throwing. (**b**) No Operating. (**c**) No Crossing.

As mentioned above, the risk of behaviors was evaluated by detecting and recognizing the safety signs in the workplace. The meaning of detected safety signs was used for judging whether the identified behavior is safe or not. If the behavior identified by the YOLO-ST-GCN method is the same as the forbidden behavior corresponding to the safety signs, it would be identified as an unsafe behavior; otherwise, it will be identified as a safe behavior. For example, if the No Throwing safety sign and throwing harmer behavior were detected in the same workplace, the behavior of throwing hammer would be identified as unsafe behavior. In this study, throwing hammer under the safety sign of No Throwing was considered unsafe behavior, while the other behaviors were considered safe behavior. Turning on switch under the safety sign of No Operating was considered as unsafe behavior, while the other behaviors were considered as safe behavior. Crossing railing under the safety sign of No Crossing was considered as unsafe behavior, while the other behaviors were considered as safe. The identification results were drawn into a confusion matrix, as shown in Figure 11. The accuracy of behavior risk evaluation considering safety signs was shown in Table 10.

For No Throwing, the overall accuracy of No Throwing was 93.30%, the accuracy of Unsafe Behavior (UB) was 85.71%, and the accuracy of Safe Behavior (SB) is 95.83%. The Unsafe Behavior (UB) was set as positive samples and Safe Behavior (SB) was set as negative samples. The evaluation indicators were calculated: $Precision=0.87,\;Recall=0.86,\;and\;F_{1}=0.86$.

For No Operating, the overall accuracy of No Operating was 99.11%, the accuracy of Unsafe Behavior (UB) was 98.21%, and the accuracy of Safe Behavior (SB) is 99.40%. The Unsafe Behavior (UB) was set as positive samples and Safe Behavior (SB) was set as negative samples. The evaluation indicators were calculated: $Precision=0.98,\;Recall=0.98,\;and\;F_{1}=0.98$.

For No Crossing, the overall accuracy of No Crossing was 100.00%, the accuracy of Unsafe Behavior (UB) was 100.00%, the accuracy of Safe Behavior (SB) was 100.00%. The Unsafe Behavior (UB) was set as positive samples and Safe Behavior (SB) was set as negative samples. The evaluation indicators were calculated: $Precision=1.00,\;Recall=1.00,\;and\;F_{1}=1.00$.

The above results show the overall accuracy was above 90.00%, the accuracy of No Operating was close to 100.00%, and No Crossing can be identified correctly completely. The above indicated that the behaviors risk evaluation by detecting and recognizing the safety signs in workplace was feasible and effective.

## 5. Discussion

At present, limited studies investigated the identification of unsafe interaction behaviors on construction sites, most of the research only focused on motion recognition, itself, which might limit its application on real construction site. This study proposed a new method of identifying construction workers’ unsafe behaviors, i.e., unsafe interaction between man–machine/material, based on ST-GCN and YOLO. Identifying the interaction between man–machine/material and evaluating the risk of behaviors by detecting and recognizing safety signs could improve the practicability of the proposed method, which could provide more direct and valuable information for safety management.

In this study, objects (hammer, switch, bottle, railing, obstacle, and safety signs) were detected by using YOLO technology, and the performance was very good (see Table 5). These results were in line with previous studies [51,52,53,54]. Moreover, YOLO models have advantages in terms of detection speed and low hardware requirements [55,56,57,58,59,60], which could be used for future real-time monitoring or deployment in lower hardware devices. For motion capture, this study utilized OpenPose technology (COCO model) to obtain time series motion data, which was used for motion identification. In this study, OpenPose had high recognition accuracy. But, when body joints were occluded by objects, the recognition of skeleton keypoints may experience a drift phenomenon. However, compared to other studies using other skeleton keypoints capture techniques (e.g., Kinect) [41,61], OpenPose performed significantly better, especially in cases with body occlusions or non-frontal tracking [62]. And in some application workplaces, the accuracy of OpenPose in capturing skeleton keypoints is not much different from traditional expensive motion analysis devices. [63]. So OpenPose was widely used in construction sites, where complex behaviors existed and the worker’s body was heavily occluded [64,65]. Therefore, YOLO and OpenPose were selected in this study and were recommended computer vision-based technologies for object identification and motion capture, respectively, at least in the application scenarios similar to this study.

The results of this study show that the performance of motion recognition only based on ST-GCN was poor. The overall identification of Throwing, Operating and Crossing was 51.79%, 61.61% and 58.04% (see Table 6 and Table 7). The reason is obvious that the motions selected in this study are quite similar. For example, there is nearly no difference in the characteristics of the motion between throwing hammer and throwing bottle, between crossing railing and crossing obstacle. Although only using ST-GCN didn’t perform well in distinguishing between similar motions in this study, it’s still a recommended technology for motion recognition in a general sense. Many previous studies utilized ST-GCN for non-similar motion recognition and found it performed well. Cao et al. [21] identified miners’ unsafe behavior (10 different types of behaviors) based on ST-GCN in their self-built dataset, with an overall identification accuracy of 86.7%. Lee et al. [65] used ST-GCN to identify 5 different unsafe behaviors of workers, with an overall identification accuracy of 87.20%. The motions in the above studies were quite different in motion characteristics.

Considering the good performance of ST-GCN in non-similar motions recognition and poor performance in similar motions recognition, this study still chose ST-GCN for motion recognition, it is just that YOLO was added and integrated, which was used for object identification. It could improve the identification accuracy of similar motions in the case when the worker performs similar motions, but the objects that interacted with the worker are different. Since, for application, those interactions are very important for judging whether the workers’ behaviors are safe or not from the standpoint of safety management. The results of this study show that compared with only using ST-GCN, the method based on YOLO-ST-GCN proposed in this paper greatly improved the identification accuracy. The overall accuracy increased from 51.79% to 85.71%, 61.61% to 99.11%, and 58.04% to 100.00%, for throwing, operating, and crossing behaviors. And, all the interactions between man–objects were well detected and identified. As mentioned above, there is limited research that integrated motion identification with objects recognition to detect interaction behaviors between man–machine/material. Liu et al. [52] studied the interaction between human and robots based on motion recognition and object recognition and found that people’s behavioral intention depends on the possession of objects, which was consistent with this study. They also used the YOLO model for object recognition, and ST-GCN with LSTM for behavior identification, and achieved good recognition results. The difference is they only used YOLO trained by a dataset of handheld objects to detect the interaction, which may achieve a poor performance in the scenario of this study.

**Figure 12:**
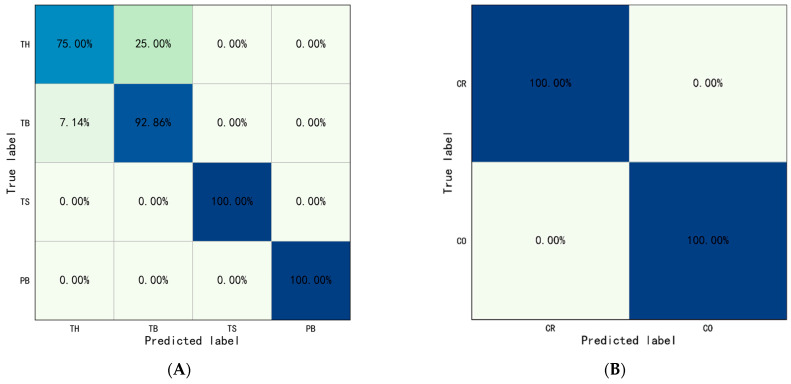
Confusion matrix of behaviors identification based on YOLO-ST-GCN based on YOLO-NAS: (**A**) Type I behaviors, (**B**) Type II behaviors. (TH: Throwing Hammer, TB: Throwing Bottle, TS: Turing on Switch, and PB: Putting Bottle).

To evaluate the effectiveness of other object detection algorithms compared to YOLOv5, we used the latest YOLO-NAS object detection algorithm. The dataset was divided randomly into a training set and a validation set in a ratio of 8:2. The batch__size was set to 8, the epoch was set to 50, and weight_decay was set to 0. 0001. The identification results were drawn into a confusion matrix, as shown in Figure 12. The comparison results of behavior identification accuracy based on YOLOv5 and YOLO-NAS were shown in Table 11.

For Type I behaviors, the results show the overall identification accuracy of Type I behaviors was 91.96%, and the overall accuracy of Throwing and Operating were 83.93% and 100.00%. The accuracy of throwing hammer, throwing bottle, turning on switch, and putting bottle were 75.00%, 92.86%, 100.00%, and 100.00%, respectively. The evaluation indicators were also calculated: $Precision=0.93,\;Recall=0.92,\;and\;F_{1}=0.92$.

For Type II behaviors, the results show the overall identification accuracy of Type II behaviors was 100.00%, the accuracy of crossing railing, and crossing obstacle were both 100.00%. The crossing railing was set as positive samples, crossing obstacle was set as negative samples. The evaluation indicators were also calculated: $Precision=1.00,\;Recall=1.00,\;and\;F_{1}-score=1.00$.

The results show that there is little difference between the accuracy of behavior identification based on YOLOv5 and YOLO-NAS. Although the latest YOLO-NAS offers state-of-the-art target detection with unmatched accuracy and speed performance, outperforming other models of the YOLO family such as YOLOv5, YOLOv6, YOLOv7, and YOLOv8 [66], the performance of using YOLOv5 is good enough for this study (i.e., interaction behavior identification based on YOLO-ST-GCN), which can meet the accuracy requirements of object recognition. There are many factors which could affect the accuracy of object recognition, e.g., occlusion of the object, low recording frame rate of the camera, and the light. The influence of these factors may outweigh the improvements in the algorithms (i.e., YOLO v5 to YOLO-NAS). For motion recognition, ST-GCN is based on the coordinates of skeleton keypoints, so accurate coordinates of skeleton keypoints are very important. However, due to the complexity of human motions and the blind field of vision of the camera, when the skeleton keypoints are occluded, the recognition results will drift. This has a certain impact on the results of behavior identification. In the future, multiple-depth cameras can be used and combined them according to certain methods to improve the accuracy of the skeleton keypoint coordinates.

**Figure 13:**
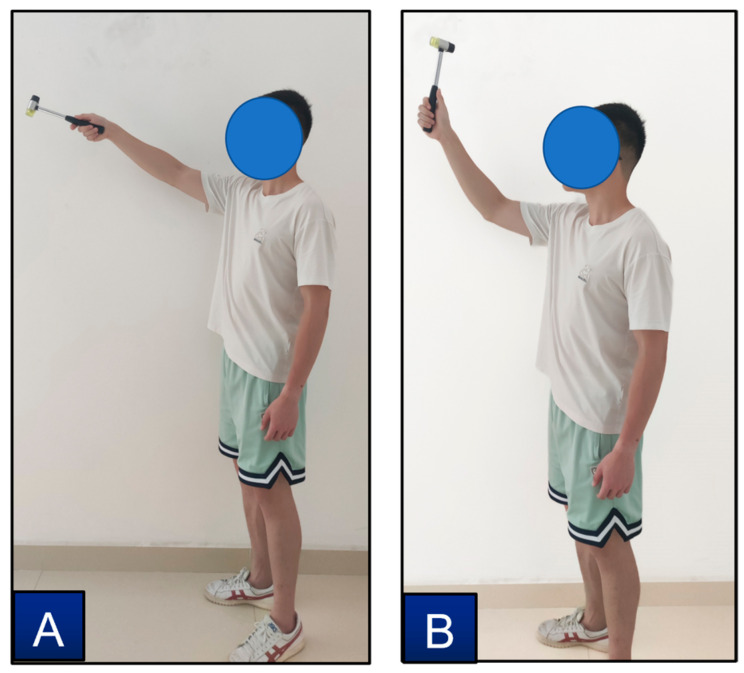
Behaviors of throwing hammer (**A**) and hammering nail (**B**).

**Figure 14:**
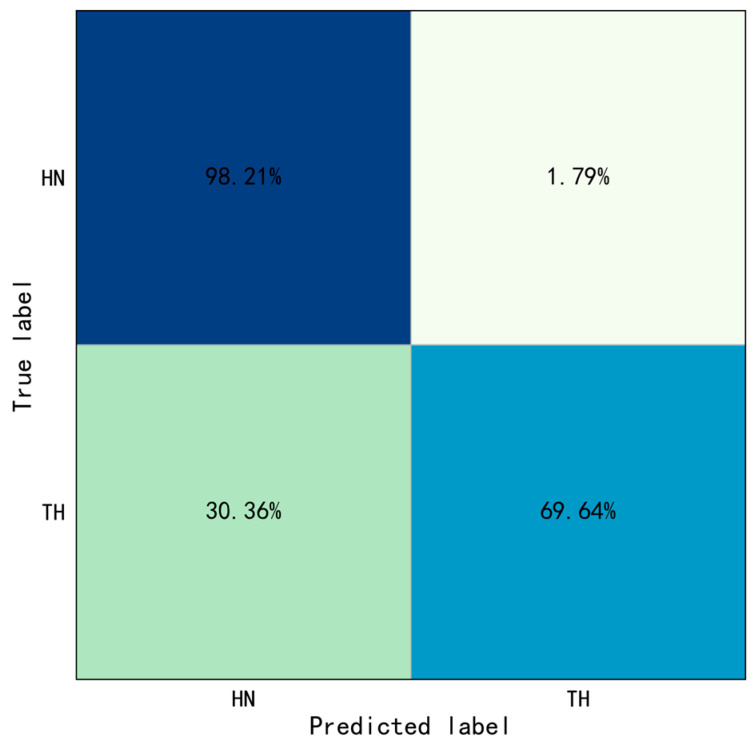
Confusion matrix of throwing hammer and hammering nail.

This study proposed the YOLO-ST-GCN method for interaction behaviors identification, the foundation was motion and object recognition. This method also has some limitations in the case that a worker performs different tasks with similar motions and interacted with the same objects. This study added one more task, hammering nail (see Figure 13B), which similar motion and same object with throwing hammer (see Figure 13A) to test the performance of the method. The behavior identification results of the confusion matrix were shown in Figure 14. The overall accuracy is 83.93%, the accuracy of hammering nail is 98.21%, and the accuracy of throwing hammer is 69.64%, the evaluation indicators were calculated: $precision=0.76,\;Recall=0.98,\;and\;F_{1}=0.86$. The results showed that 30.36% of throwing hammer were misidentified as hammering nail. Therefore, caution should be taken when using the proposed method for some cases like the above.

The limitations of the research need to be acknowledged. Firstly, a more completed dataset for training and testing the models is expected, Since, a more completed dataset that covers more work tasks, different scenarios, different angles, and different lighting conditions could improve its application to real construction sites. Secondly, the experimental tasks (i.e., behaviors in Table 2) were selected based on the field studies, but the participants in this study were recruited from a convenience sample, not the real construction workers. Thirdly, there still were limitations of the proposed method, as discussed in the above paragraph, and this study did not overcome it.

## 6. Conclusions

This study developed a new method of identifying construction workers’ unsafe interaction behaviors, i.e., unsafe interaction between man–machine/material, based on ST-GCN and YOLO. The research achieved the following findings. Firstly, YOLO, OpenPose, and ST-GCN performed well in object detection, motion capture and motion recognition, respectively. In addition, compared with object recognition, motion recognition is more susceptible to many factors. Therefore, the choice of motion recognition technology is particularly important. Secondly, the experimental tasks (i.e., behaviors in Table 1) were selected based on the field studies, but the participants in this study were not real construction workers and were recruited from a convenience sample. Thirdly, detecting and extracting the meaning of safety signs, which was used for the behaviors risk evaluation, was convenient and effective, especially for computer vision-based intelligent systems. The findings of the study have some practical implications for safety management, especially workers’ behavior monitoring and management. It could overcome the problem that the interaction behaviors are difficult to detect and diagnose on construction sites, where the workers’ behaviors and interacted objects are quite complex. In addition, more attention should be paid to applying the proposed method to identifying the behaviors with similar motions and interacting with the same or similar objects.

## Figures and Tables

**Table 1:** Overview of main characteristics of related studies.

Study	Category	Interaction Considered or Not	Technology Used	Object Identified	Performance	Limitations
Cao et al., 2023 [21]	Motion recognition	No	Improved ST-GCN	Mining behaviors	Recognition accuracy on NTU-RGB + D and self-built data sets were 94.7% and 94.1%, respectively	Some behaviors samples of dataset are too few
Yu et al., 2017 [41]	Motion recognition	No	Kinect	Leaning on a handrail, Dumping from height, Climbing	Total accurate rate was up to 81.44%	Inadequate feature parameters.
Franco et al., 2020 [42]	Motion recognition	No	Kinect	Three public datasets CAD 60, CAD 120, OAD	Precision was 98.8%, 85.4%, and 90.6%, respectively	Lack of explicit modeling of user interaction with objects
Ding et al., 2018 [43]	Motion recognition	No	CNN + LSTM	Four types of ladders climbing motions	Accuracy in recognizing all four types of motions was 92%	Lack of data set and cannot be able to determine the relationships between equipment and workers
Fang et al., 2019 [17]	Object recognition	No	Mask R-CNN	People who traverse concrete/steel supports	Recall and precision rates were 90% and 75%, respectively	The method depends on the overlapping area to judge the safety, which is easy to misidentify
Hu et al., 2022 [44]	Object recognition	No	Faster R-CNN	Throwing, lying, relying, jumping, and without helmets	Accuracy and precision were 93.46% and 99.71%, respectively	Identifying unsafe behaviors by recognize human, which requires a large dataset
Fang et al., 2018 [45]	Object recognition	No	Faster R-CNN	Workers wear or not wear harness	Precision and recall rates were 99% and 95%, respectively	The dataset was too small, and only selected a few activities
Zhang et al., 2022 [40]	Interaction recognition	YES	Mask RCNN and BERT	Nine types of construction components and seven types of interactions.	Identification accuracy was 97.82%	Pre-tasks are complex and time-consuming

**Table 2:** Examples of unsafe behaviors selected in this study.

Type	Description	Examples of Behaviors	
I	Unsafe contact between man-machine/material	Throwing	Throwing Hammer (TH)
			Throwing Bottle (TB)
		Operating	Turning on Switch (TS)
			Putting Bottle (PB)
II	Unsafe distance to machine/material (no physical contact)	Crossing	Crossing Railing (CR)
			Crossing Obstacle (CO)

**Table 3:** Configuration parameters.

Device	Configuration
Operating system	Windows 11 (64-bit)
CPU	AMD Ryzen 7 4800H with Radeon Graphics 2.90 GH
RAM	32 G
GPU	NVIDIA RTX2060, 6 G
GPU accelerator	Cuda 11.3
Framework	Pytorch 1.8.1
Scripting language	Python 3.8

**Table 4:** Number of videos of each behavior.

Behaviors		Number
Throwing	Throwing Hammer (TH)	14 × 20 × 3 *
	Throwing Bottle (TB)	14 × 20 × 3
Operating	Turning on switch (TS)	14 × 20 × 3
	Putting Bottle (PB)	14 × 20 × 3
Crossing	Crossing Railing (CR)	14 × 20 × 3
	Crossing Obstacle (CO)	14 × 20 × 3

* 14: 14 participants. 20: Two cameras recorded multiply repeating 5 times with their left and right hands. 3: Three workplaces for pasting safety signs.

**Table 5:** YOLO training results for the object detection.

Class		Precision	Recall	mAP@0.5
Objects	Bottle	0.995	1	0.995
	Hammer	0.998	1	0.995
	Switch	0.998	1	0.995
	Railing	0.994	1	0.995
	Obstacle	0.997	1	0.994
Safety signs	No Throwing	0.998	1	0.995
	No Operating	0.999	1	0.995
	No Crossing	0.998	1	0.995

**Table 6:** Identification accuracy of Type I behaviors only based on ST-GCN.

Behaviors		Accuracy		
Throwing	Throwing Hammer (TH)	89.29%	51.79%	56.70%
	Throwing Bottle (TB)	14.29%		
Operating	Turning on switch (TS)	62.50%	61.61%	
	Putting Bottle (PB)	60.71%		

**Table 7:** Identification accuracy of Type II behaviors only based on ST-GCN.

Behaviors		Accuracy	
Crossing	Crossing Railing (CR)	71.43%	58.04%
	Crossing Obstacle (CO)	44.64%	

**Table 8:** Identification accuracy of Type I behaviors based on YOLO-ST-GCN.

Behaviors		Accuracy		
Throwing	Throwing Hammer (TH)	85.71%	85.71%	92.41%
	Throwing Bottle (TB)	85.71%		
Operating	Turing on Switch (TS)	98.21%	99.11%	
	Putting Bottle (PB)	100.00%		

**Table 9:** Identification accuracy of Type II behaviors based on YOLO-ST-GCN.

Behaviors		Accuracy	
Crossing	Crossing Railing (CR)	100.00%	100.00%
	Crossing Obstacle (CO)	100.00%	

**Table 10:** Identification accuracy of behaviors risk evaluation considering safety signs identification.

Safety Signs	Behavior	Accuracy	
No Throwing	Unsafe Behavior (UB)	85.71%	93.30%
	Safe Behavior (SB)	95.83%	
No Operating	Unsafe Behavior (UB)	98.21%	99.11%
	Safe Behavior (SB)	99.40%	
No Crossing	Unsafe Behavior (UB)	100.00%	100%
	Safe Behavior (SB)	100.00%	

**Table 11:** Comparison in the results of behavior identification accuracy.

Behaviors		Accuracy Base on YOLOv5			Accuracy Base on YOLO-NAS		
Throwing	Throwing Hammer (TH)	85.71%	85.71%	92.41%	75.00%	83.93%	91.96%
	Throwing Bottle (TB)	85.71%			92.86%		
Operating	Turing on Switch (TS)	98.21%	99.11%		100.00%	100.00%	
	Putting Bottle (PB)	100.00%			100.00%		
Crossing	Crossing Railing (CR)	100.00%	100.00%		100.00%	100.00%	
	Crossing Obstacle (CO)	100.00%			100.00%		

## Data Availability

The date can be obtained from the corresponding author upon reasonable request.
